# Supplementary material for: A mitogenomic timetree for Darwin’s enigmatic South American mammal Macrauchenia patachonica
Source: Nat Commun. 2017 Jun 27;8:15951. doi: 10.1038/ncomms15951 (PMC5490259; doi:10.1038/ncomms15951)
Supplement: Supplementary Information [file ncomms15951-s1.pdf]

Type of file: PDF
Size of file: 0 KB
Title of file for HTML: Supplementary Information
Description: Supplementary Figures, Supplementary Tables, Supplementary Note and Supplementary References

Type of file: PDF
Size of file: 0 KB
Title of file for HTML: Peer Review File
Description:

Supplementary Figures

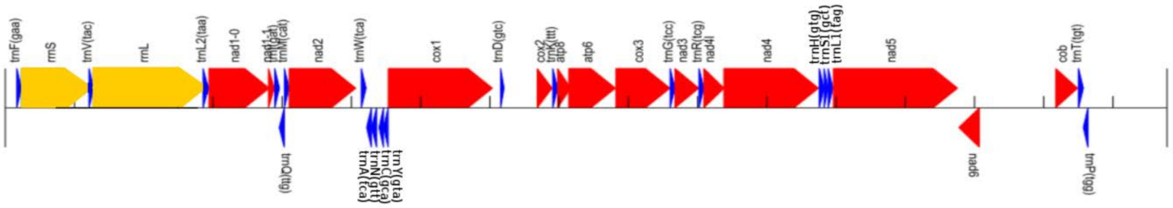

Supplementary Figure 1. MITOS output. Protein coding gene (red), tRNA (blue) and rRNA (yellow) presence and approximate location along the reconstructed MAC002 mitochondrion.

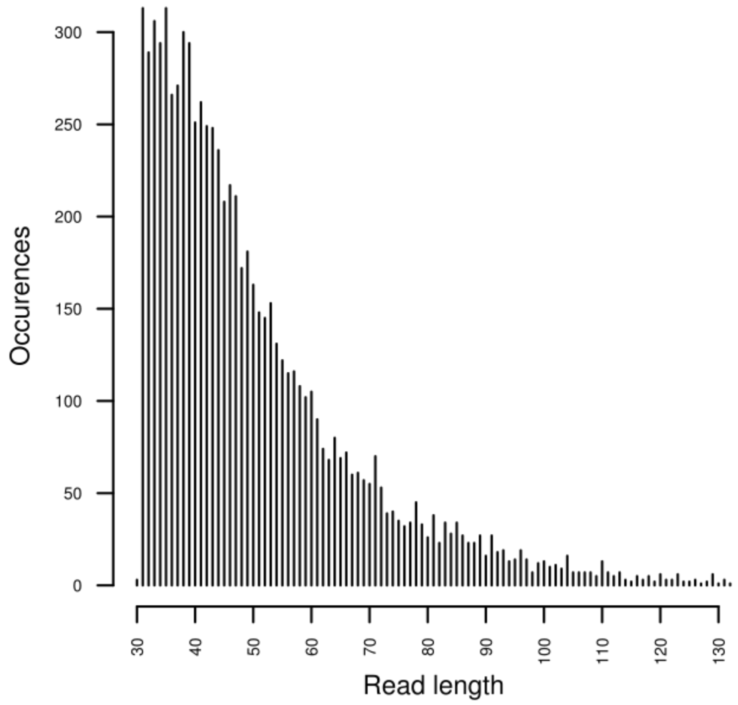

Supplementary Figure 2. Read length Mapdamage output. Read length distribution of MAC002 reads mapped to our *Macrauchenia* mitochondrial sequence.

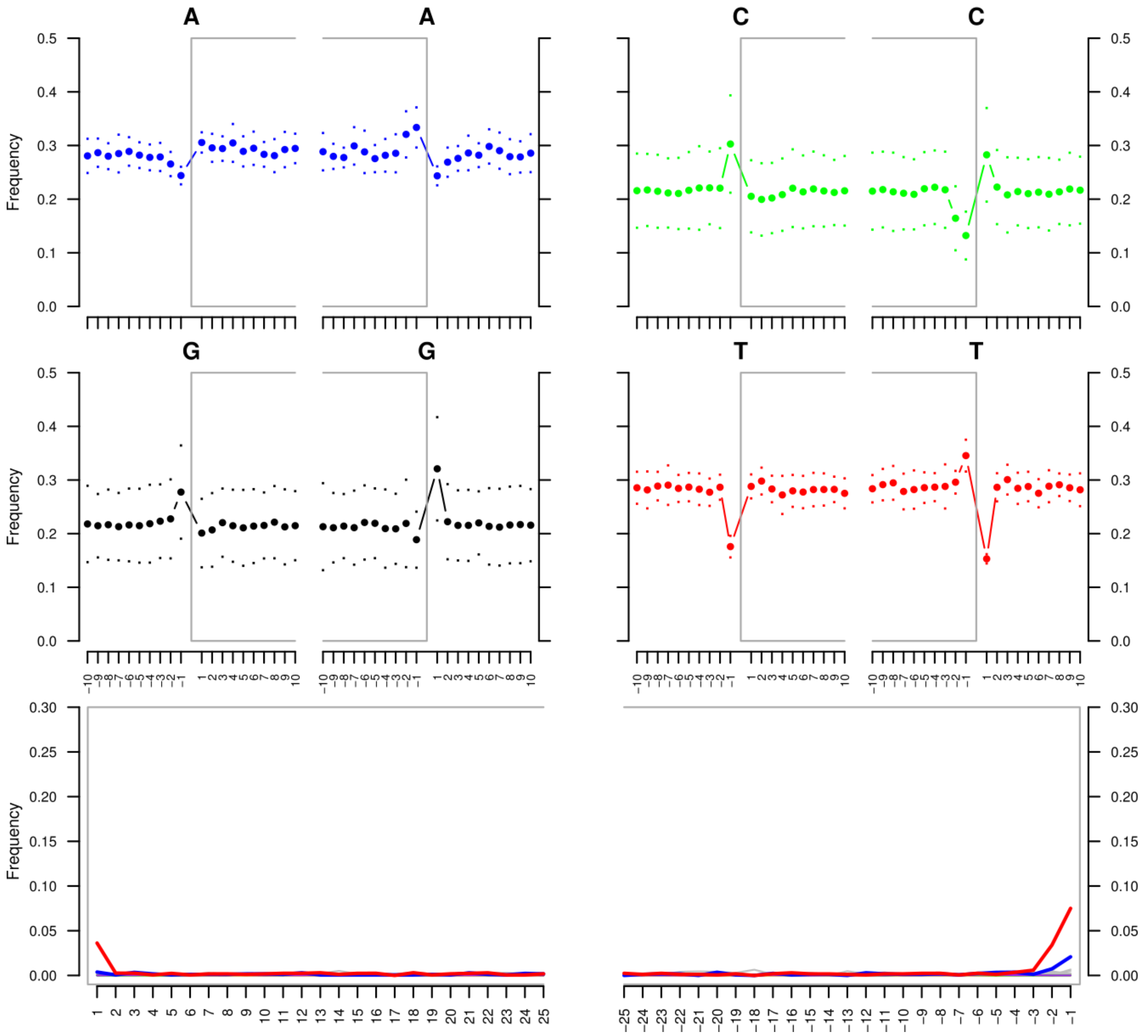

Supplementary Figure 3: Damage pattern Mapdamage results. There is an increased number of T bases (indicated in red) at the ends of reads when reads were mapped back to our MAC002 *Macrauchenia* mitochondrial sequence. X axis represents position from 5' (left) and 3' (right) read end.

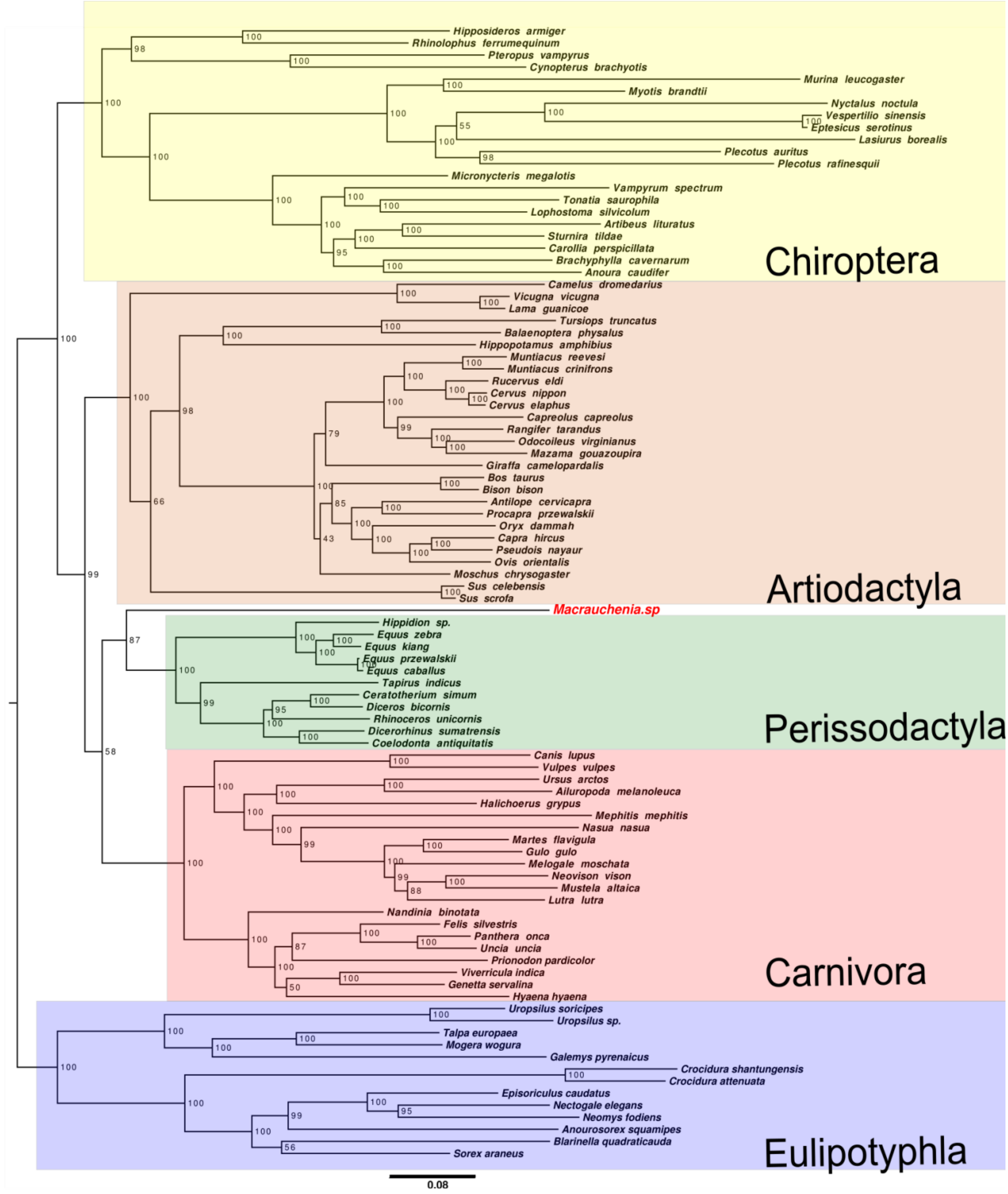

Supplementary Figure 4. Maximum likelihood mitochondrial genome tree for our complete dataset consisting of the *Macrauchenia* and selected representatives of the Laurasiatheria superorder. Numbers at nodes represent bootstrap values. *Macrauchenia* is sister to the Perissodactyla clade. Scale bar indicates substitutions per site.

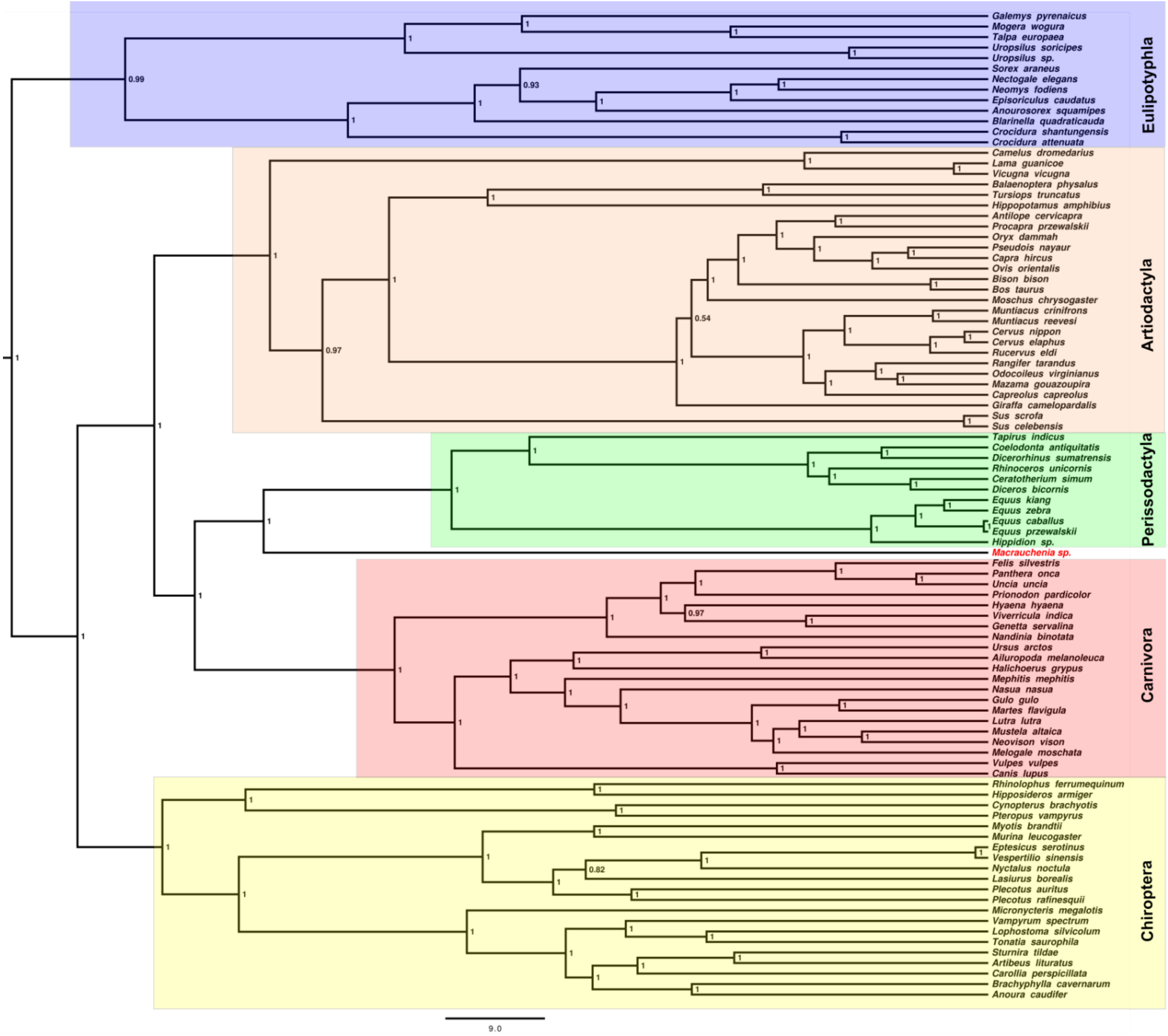

Supplementary Figure 5. Bayesian mitochondrial genome tree for our complete dataset consisting of the *Macrauchenia* and selected representatives of the Laurasiatheria superorder. Posterior clade probabilities are indicated on nodes. Scale bar represents millions of years.

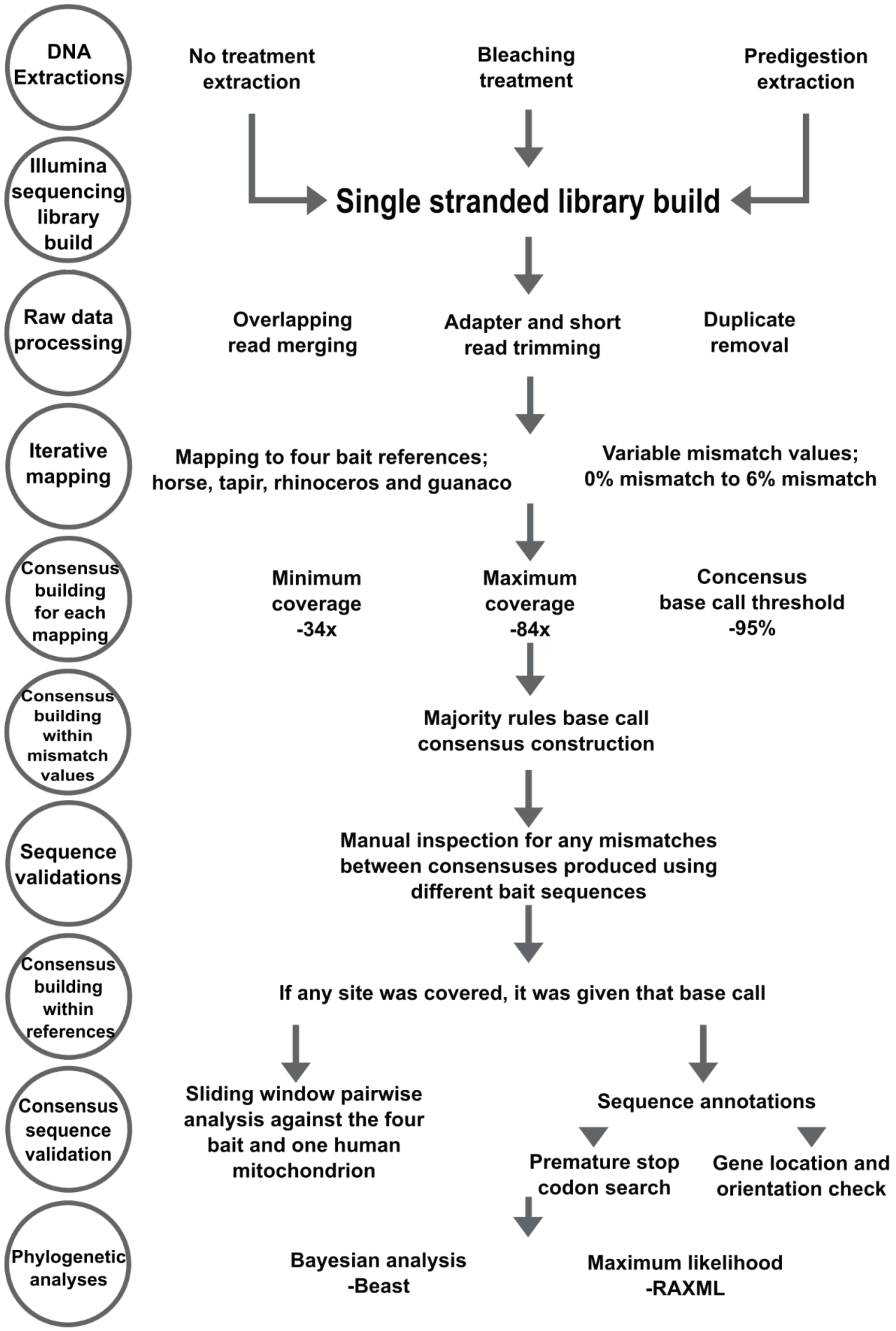

Supplementary Figure 6. Diagram presenting the work-flow of methods used to construct and analyse the mitochondrial genome of MAC002.

Supplementary Tables

Supplementary Table 1. Details of *Macrauchenia* and *Toxodon* samples used in this report.

Collection No.	Sample ID	Species	Locality	Latitude (S); longitude (W)	Material
GCF-1	TOX2	*Toxodon platensis*	Campo Spósito, San Pedro, Argentina	33°44'; 59°36'	petrosal
MACN-PV 5712	TOX005	*Toxodon platensis*	Tapalqué, Buenos Aires, Argentina	36°21'; 60°01' *	Phalanx & carpal bone
MACN-PV 5718	TOX008	*Toxodon platensis*	Tapalqué, Buenos Aires, Argentina	36°21'; 60°01' *	petrosal
MACN-PV 11382	TOX007	*Toxodon platensis*	Carcarañá, Santa Fe, Argentina	32°51'; 61°09' *	tibia
MACN-PV 11527	TOX1	*Toxodon platensis*	Carcarañá, Santa Fe, Argentina	32°51'; 61°09' *	petrosal
MACN-PV 14110	TOX009	*Toxodon platensis*	Argentina, old collections	NA	humerus
MACN-PV 17710	TOX004	*Toxodon platensis*	Tapalqué, Buenos Aires, Argentina	36°21'; 60°01' *	tibia
MLP 12-1174	TOX001	*Toxodon platensis*	Arrecifes, Buenos Aires, Argentina	34°04'; 60°07' *	petrosal
MNHN(U) 150	TOX002	*Toxodon platensis*	South bank of Río Negro River, 5ta Sección, Dto Durazno, Uruguay	33°22'; 56°31' *	petrosal
MNHN(U) 379	TOX003	*Toxodon platensis*	Departomento Colonia, Uruguay	34°28'; 57°50' *	humerus
MNHN(U) no number	TOX006	*Toxodon platensis*	Uruguay, old collections	NA	humerus
MMP 5019-M	RAU2	*Macrauchenia patachonica*	Camet Norte, Buenos Aires, Argentina	37°49'; 57°29'	mandible
MNHN-F-TAR 817	RAU1	*Macrauchenia patachonica*	Tarija, Bolivia	21°32'; 64°44'	Metatarsal
UISEK/KM/No number	MAC001	*Macrauchenia patachonica*	Kamac Mayu, Calama, Chile	22°28'; 68°56'	Molar root
UISEK/KM/B2/3	MAC003	*Macrauchenia patachonica*	Kamac Mayu, Calama, Chile	22°28'; 68°56'	Metapodial
UISEK/KM/No number	MAC004	*Macrauchenia patachonica*	Kamac Mayu, Calama, Chile	22°28'; 68°56'	Petrosal
FACSO/BN-1/2A/5	MAC002	*Macrauchenia patachonica*	Baño Nuevo-1 Cave, Coyhaique, Chile	45°17'; 71°32'	Middle phalanx

*As specific localities are rarely indicated in older collections, coordinates are of nearest town

Institutional abbreviations:
FACSO/BN, Facultad de Ciencias Sociales, Universidad de Chile, Baño Nuevo-1 collection, Santiago, Chile
UISEK/KM, Universidad Internacional SEK-Chile, Kamac Mayu collection, Santiago, Chile
MACN-PV, Museo Argentino de Ciencias Naturales, vertebrate paleontology collection, Buenos Aires, Argentina
MNHN(U), Museo Nacional de Historia Natural, Montevideo, Uruguay
MLP, Museo de La Plata, vertebrate paleontology collection, Buenos Aires, Argentina
MNHN-F-TAR, Musee National de Histoire Natural, Tarija collection, Paris, France

Supplementary Table 2. ***Macrauchenia*** **and** ***Toxodon*** **test sequencing and mapping results.**

Given sample code name	Number of raw reads	Total number of read pairs after trimming and adapter removal	Number of combined read pairs	Number of reads uniquely mapped to horse	% of merged, trimmed reads mapping to horse	% of raw read pairs mapping to horse	Number of reads uniquely mapped to rhino	% of merged, trimmed reads mapping to rhino	% of raw read pairs mapping to rhino
**MAC001**	3105006	1699322	1646515	5896	0.3580896621	0.1898869117	7733	0.4696586426	0.249049438
**MAC002**	1934987	1560104	1512035	36822	2.44	1.9	41992	2.78	2.17
**MAC002 (bleach) ***	2303144	1668567		26311	1.576862062	1.142394918	30904	1.852128203	1.341817967
**MAC002 (predigest) ***	3470116	2609499		37138	1.423185063	1.070223589	43466	1.665683719	1.252580605
**MAC003**	3002543	1197249	1166376	6235	0.5345617537	0.2076573092	7992	0.6851992839	0.266174372
**MAC004**	3018104	1362016	1326751	7652	0.576747257	0.2535366575	10067	0.7587708621	0.333553780
**TOX008***	2599640	1422642		1961	0.1378421275	0.0754335216	2382	0.1674349555	0.091628071
**TOX009***	2609664	1529771		816	0.0533413171	0.0312683932	996	0.0651077841	0.038165832
**RAU1**	19932093	18360464	7373163	986	0.0053702347	0.0049467961	1066	0.0058059535	0.005348158
**RAU2**	11191261	10305156	4997463	680	0.0065986386	0.0060761696	717	0.0069576822	0.006406784
**TOX1**	7023066	6112488	4448756	68	0.0011124766	0.0009682381	92	0.0015051154	0.001309969
**TOX2**	8058659	7833419	2275358	427	0.0054510042	0.0052986483	428	0.00546377	0.005311057
**TOX001**	6493167	6106757	2047118	2347	0.0384328376	0.0361456898	2629	0.0430506732	0.040488716
**TOX002**	6717421	6055758	3905117	179	0.0029558645	0.0026647131	197	0.0032531023	0.002932673
**TOX003**	6506929	5812021	3959820	27476	0.4727443345	0.4222575657	13804	0.2375077447	0.212143086
**TOX004**	10062032	9272949	4390415	176	0.0018979938	0.0017491497	187	0.0020166184	0.001858471
**TOX005**	2910385	2692588	1463995	247	0.0091733306	0.0084868497	255	0.0094704426	0.008761727
**TOX006**	7729600	7274714	2919959	177	0.0024330853	0.0022898986	85	0.0011684308	0.001099668
**TOX007**	7900849	7419317	3819959	112	0.0015095729	0.0014175692	112	0.0015095729	0.001417569

* Note: sequenced using single ended reads

Supplementary Table 3. Pairwise distance comparisons between consensus sequences from different bait sequences when using MITObim default parameters.

Bait reference 1	Bait reference 2	Pairwise distance
Horse	Rhino	0.16
Horse	Guanaco	0.43
Rhino	Guanaco	0.42

Supplementary Table 4. Comparisons of number of mismatches between MITObim produced cave hyena mitochondrial sequence and the sequence produced using BWA when using different minimum coverage cutoffs for consensus calling.

Mismatch %	MITObim bait reference	Number of sites of mitogenome covered	Number of mismatches at 1x coverage consensus calling compared to bwa sequence	Number of mismatches 80% of the average coverage compared to bwa sequence
0	Brown bear	1393	25	0
1	Brown bear	6121	51	0
2	Brown bear	6361	53	0
6	Brown bear	7016	24	0
10	Brown bear	7611	24	0
12	Brown bear	6931	24	0
0	Dog	3985	27	0
1	Dog	7223	17	0
2	Dog	7586	18	0
6	Dog	7430	42	0
10	Dog	7571	5	0
12	Dog	7567	9	0

Supplementary Table 5. Percentages of mitochondrial genome covered when using different mismatch values and bait reference sequences.

Bait reference	mismatch value	% of the mitogenome covered
Horse	0	23.2
Horse	1	52.8
Horse	2	49.0
Horse	3	50.5
Horse	4	54.7
Horse	5	58.0
Horse	6	60.4
Tapir	0	23.6
Tapir	1	58.0
Tapir	2	62.4
Tapir	3	61.1
Tapir	4	69.6
Tapir	5	66.6
Tapir	6	67.9
Rhinoceros	0	14.1
Rhinoceros	1	32.6
Rhinoceros	2	36.2
Rhinoceros	3	40.1
Rhinoceros	4	41.2
Rhinoceros	5	39.9
Rhinoceros	6	66.9
Guanaco	0	10.1
Guanaco	1	10.1
Guanaco	2	10.1
Guanaco	3	50.6
Guanaco	4	50.7
Guanaco	5	67.2
Guanaco	6	74.6

Supplementary Table 6. Estimated *Macrauchenia* (Panperissodactyla) divergence dates based on different fossil calibrations.

Calibration node	Mean divergence time (MYA)	95% CI upper limit	95% CI lower limit
Crown Laurasitheria	54.82	75.82	40.22
Crown Carnivore	48.91	61.61	38.99
Crown Bovidae	55.37	72.48	41.76
Crown Perissodactyla	78.82	94.51	63.09
Combination of the above four	66.15	77.83	56.64

Supplementary Table 7. Estimated divergence times for each major clade using the combination of the four calibration points described in Supplementary Table 6.

Clade	Mean (MYA)	Lower 95% CI (MYA)	Upper 95% CI (MYA)
Perissodactyla	48.88	47.8	51.00
Carnivora	54.09	45.16	64.92
Artiodactyla	65.51	54.66	78.08
Panperissodactyla	66.15	56.64	77.83
Chiroptera	75.48	63.46	89.02
Eulipotyphla	78.82	63.06	95.92
Laurasiatheria	89.19	73.88	104.62

Supplementary Table 8. *Macrauchenia* and *Toxodon* samples that received pretreatment with bleach.

Given code name	Sample details
MAC001	Molar root Kamac mayu(Calama) Grid B2/Layer:3 Fondecyt Integracion Nueva Calama
MAC002	2nd phalanx Bano nuevo-1 (Coyhaique) Grid 2A/Layer:5 Fondecyt 1030560
MAC003	Metapodial Kamac mayu(Calama) Grid B2/Layer:3 Fondecyt Integracion Nueva Calama
MAC004	Petrosal Kamac mayu (Calama) Grid: 0 Layer 0 Fondecyt Integracion Nueva Calama
TOX008	MACN 14110
TOX009	MACN 5718

Supplementary Table 9. Total number of MAC002 reads remaining after various control stages.

	Number of reads
Raw paired end reads	68891960
PE reads post-duplicate removal	65268335
Post adapter, low quality and short read trimming	43917870
Post PE merging	42928963

Supplementary Table 10. Species names and Genbank accession numbers of mitochondrial sequences used in the multiple sequence alignment.

**Acession code**	**Genus**	**species**
GU946995	*Bison*	*bison*
NC004577	*Muntiacus*	*crinifrons*
HQ832482	*Cervus*	*nippon*
JQ608470	*Moschus*	*chrysogaster*
KJ772514	*Mazama*	*gouazoupira*
KM612279	*Odocoileus*	*virginianus*
KF312238	*Ovis*	*orientalis*
KJ681486	*Capreolus*	*capreolus*
KP172593	*Cervus*	*elaphus*
KP662715	*Capra*	*hircus*
KM506758	*Rangifer*	*tarandus*
NC024860	*Sus*	*celebensis*
JX101652	*Pseudois*	*nayaur*
JN869311	*Oryx*	*dammah*
NC014701	*Rucervus*	*eldi*
NC014875	*Procapra*	*przewalskii*
NC012100	*Giraffa*	*camelopardalis*
NC012098	*Antilope*	*cervicapra*
NC009849	*Camelus*	*dromedarius*
EF035447	*Muntiacus*	*reevesi*
KC572860	*Balaenoptera*	*physalus*
NC027237	*Nyctalus*	*noctula*
NC026465	*Cynopterus*	*brachyotis*
NC025949	*Murina*	*leucogaster*
NC024558	*Vespertilio*	*sinensis*
NC018540	*Hipposideros*	*armiger*
NC016872	*Plecotus*	*rafinesquii*
NC016871	*Artibeus*	*lituratus*
JN209842	*Lasiurus*	*borealis*
NC022474	*Eptesicus*	*serotinus*
NC022429	*Vampyrum*	*spectrum*
NC022428	*Tonatia*	*saurophila*
NC022427	*Sturnira*	*tildae*
NC022424	*Lophostoma*	*silvicolum*

NC022422	*Carollia*	*perspicillata*
NC022421	*Brachyphylla*	*cavernarum*
NC022420	*Anoura*	*caudifer*
NC022419	*Micronycteris*	*megalotis*
NC015484	*Plecotus*	*auritus*
HQ685964	*Ursus*	*arctos*
KM488625	*Neovison*	*vison*
KM347744	*Martes*	*flavigula*
NC025296	*Viverricula*	*indica*
NC021751	*Mustela*	*altaica*
NC024568	*Genetta*	*servalina*
NC024567	*Nandinia*	*binotata*
KJ636050	*Prionodon*	*pardicolor*
KF387633	*Vulpes*	*vulpes*
EF672696	*Lutra*	*lutra*
KM236783	*Panthera*	*onca*
KR611313	*Gulo*	*gulo*
NC020648	*Mephitis*	*mephitis*
NC001602	*Halichoerus*	*grypus*
EF551004	*Uncia*	*uncia*
HM106331	*Nasua*	*nasua*
HM106328	*Melogale*	*moschata*
EF196663	*Ailuropoda*	*melanoleuca*
KP202275	*Felis*	*silvestris*
KF926377	*Bos*	*taurus*
FJ905816	*Dicerorhinus*	*sumatrensis*
NC020433	*Equus*	*kiang*
NC020476	*Equus*	*zebra*
NC002008	*Canis*	*lupus*
NC012059	*Tursiops*	*truncatus*
NC011822	*Lama*	*guanicoe*
KM881677	*Hippidion*	*sp.*
NC000889	*Hippopotamus*	*amphibius*
EU939445	*Equus*	*caballus*
KT368758	*Equus*	*przewalskii*
NC020669	*Hyaena*	*hyaena*
JX034737	*Uropsilus*	*sp.*
NC026204	*Crocidura*	*attenuata*
NC026131	*Episoriculus*	*caudatus*
NC025559	*Neomys*	*fodiens*
KC503902	*Nectogale*	*elegans*
NC023244	*Uropsilus*	*soricipes*
NC021398	*Crocidura*	*shantungensis*
NC023950	*Blarinella*	*quadraticauda*
NC002391	*Talpa*	*europaea*
NC005035	*Mogera*	*wogura*
AY833419	*Galemys*	*pyrenaicus*
NC024563	*Anourosorex*	*squamipes*

NC025308	*Myotis*	*brandtii*
KP126954	*Sus*	*scrofa*
Y07726	*Ceratotherium*	*simum*
NC016191	*Rhinolophus*	*ferrumequinum*
NC012682	*Diceros*	*bicornis*
NC001779	*Rhinoceros*	*unicornis*
NC012681	*Coelodonta*	*antiquitatis*
NC027963	*Sorex*	*araneus*
KJ417810	*Tapirus*	*indicus*
KP214033	*Pteropus*	*vampyrus*
FJ456892	*Vicugna*	*vicugna*

Supplementary Table 11. Genes and RNA sequences associated with each partition used in the Raxml analysis.

partition number	tRNA and gene in partition
1	tRNA-Ile, tRNA-Leu, tRNA-Met2, tRNA-Pro, tRNA-Ser2
2	ATP8, ND2
3	tRNA-Asp, tRNA-Gly, tRNA-His, tRNA-Leu2, tRNA-Lys, tRNA-Ser, tRNA-Trp, tRNA-Tyr, tRNA-Val
4	12S, tRNA-Ala, tRNA-Arg, tRNA-Phe, tRNA-Thr
5	16S, tRNA-Asn, tRNA-Met1
6	TRNA-Cys
7	COX1
8	ATP6, COX2, COX3, ND3, ND4L, tRNA-Gln
9	ND4, ND5, ND6, tRNA-Glu
10	CYTB, ND1

Supplementary Table 12. Genes and RNA sequences associated with each partition along with the substitution model associated with each partition for the BEAST analysis.

partition number	tRNA and gene in partition	Substitution models
1	COX1, tRNA-Met2	GTR I+G
2	ND2, tRNA-Glu	GTR I+G
3	12S, tRNA-Ala, tRNA-Gly, tRNA-His, tRNA-Leu2, tRNA-Lys, tRNA-Phe, tRNA-Ser, tRNA-Thr, tRNA-Trp, tRNA-Tyr, tRNA-Val	GTR I+G
4	16S, tRNA-Asn, tRNA-Met1	GTR I+G
5	tRNA-Cys	SYM+G
6	tRNA-Arg, tRNA-Asp, tRNA-Ile, tRNA-Leu, tRNA-Ser2	GTR I+G
7	ATP6, COX2, COX3, ND3, ND4L, tRNA-Gln, tRNA-Pro	GTR I+G
8	ATP8, ND4, ND5, ND6	GTR I+G
9	CYTB, ND1	GTR I+G

Supplementary Table 13. Species considered as ingroup for each fossil calibration analysis.

Ingroup species when using crown Bovidae fossil calibration	Ingroup species when using crown Laurasiatheria fossil calibration	Ingroup species when using crown Carnivora fossil calibration	Ingroup species when using crown Perissodactyla fossil calibration
Antilope cervicapra	All species in the alignment	*Ailuropoda melanoleuca*	*Equus kiang*
Bison bison		*Canis lupus*	*Equus zebra*
Bos taurus		*Felis silvestris*	*Equus cabullus*
Capra hircus		*Genetta servalina*	*Equus przewalskii*
Oryx dammah		*Gulo gulo*	*Tapirus indicus*
Ovis orientalis		*Halichoerus grypus*	*Coelodonta antiquitatis*
Procapra przewalskii		*Hyaena hyaena*	*Dicerorhinus sumatrensis*
Pseudois nayaur		*Lutra lutra*	*Rhinoceros unicornis*
		Martes flavigula	*Ceratotherium simum*
		Melogale moschata	*Diceros bicornis*
		Mephitis mephitis	*Hippidion sp.*
		Mustela altaica	
		Nandinia binotata	
		Nasua nasua	
		Neovison vison	
		Panthera onca	
		Prionodon pardicolor	
		Uncia uncia	
		Ursus arctos	
		Viverricula indica	
		Vulpes vulpes	

Supplementary Note 1: Systematic Context of *Macrauchenia patachonica*

Overview of litoptern systematics. Litopterns were cursorial herbivores with mesaxonic limbs and several unusual cranial and dental features. In distinction to most other SANUs, litopterns primitively retained rooted teeth and developed varied cheektooth morphologies, including bunodonty (*e.g.*, Megadolodinae), bunolophodonty (*e.g.*, Proterotheriinae) and lophoselenodonty (*e.g.*, Macraucheniidae)[1,2,3]. As the result of continuing discovery over many decades, we now know that litopterns were present in South America for most of the Cenozoic. One group (Sparnotheriodontidae) even reached West Antarctica when this was connected to southernmost South America prior to the appearance of the Drake Passage[4,5.6].

Unquestionable litopterns are traditionally classified in three families, Adianthidae, Proterotheriidae, and Macraucheniidae. The latter two were taxically dominant, with macraucheniids persisting from the Late Eocene (Mustersan South American Land Mammal Age or SALMA) through to the end of the Pleistocene (Lujanian, local Stage/Age)[7,8]. Other, mostly Paleogene groups have a less certain connection with these core litoptern families. Anisolambdidae, Notonychopidae, Indalecidae, Protolipternidae, Amilnedwardsiidae, Sparnotheriodontidae and even archaic ungulates such as Didolodontidae have traditionally been included within the order (*e.g.*, refs.[1,9-11]), but their positions have been repeatedly questioned (*e.g.*, refs.[12-15]). Among these putative litopterns the oldest known is *Requisia vidmari*, from the Early Paleocene locality of Punta Peligro in central Patagonia[10,16], currently dated to 63.2 – 63.8 Ma[17]. Isolated astragali from the Early Eocene locality of Itaboraí (eastern Brazil) resemble those of definite proterotheriids, leading some authors to speculate that this clade was already in existence by this time (*e.g.*, refs. [14,18-20]). However, proterotheriid dentitions are first recorded in the Late Oligocene, during the Deseadan SALMA[3].

Late Eocene (Mustersan SALMA) *Polymorphis*, the oldest known definite macraucheniid[14], serves to date the origin of the family paleontologically. Differing from the horse-like proterotheriids, macraucheniids had a robust body structure more like that of modern camels, featuring three-toed autopodia, a long neck, and reduced nasals. In Late Miocene–Pleistocene macraucheniines, some of these features evolved in bizarre directions, as in the case of the retraction of the nasal aperture[21]. From a position at the end of the rostrum, as in typical mammals, this opening progressively moved dorsally. Pleistocene *Macrauchenia*, in which the nasal aperture is perched between the orbits, near the summit of the skull, represents a morphological extreme. It has long been speculated whether so drastic a modification implies that the snout was elaborated into a proboscis[8,22-25].

Macraucheniidae includes either two or three subfamilies, depending on whether Theosodontinae is separately recognized. Macraucheniinae and Cramaucheniinae contain most of the approximately 18 genus-level taxa currently recognized for the family (ignoring possible synonyms). Progressive increases in body size, cheektooth crown height, and snout length strongly marked the evolution of Macraucheniinae[1,21, 23,24,26-28]. From the standpoint of taxonomic richness,

macraucheniine peak diversity was achieved in the Late Miocene. They became extinct early in the Holocene, *Macrauchenia* itself being the last surviving taxon (last appearance date: 8390 ± 140 14C yr BP[8,29]). *Macrauchenia* (and its close relatives *Macraucheniopsis* and *Xenorhinotherium*) had a broad distribution across the continent, although as in the case of other South American native ungulates known fossils overwhelmingly come from the southern cone (Supplementary fig. 7, 8). Unlike toxodontid notoungulates, which managed to penetrate Central and southern North America after the completion of the Isthmus of Panama, *Macrauchenia* and other litopterns failed to participate in the Great American Biotic Interchange.

History of classification and phylogenetic relationships. The relationships of litopterns and other SANUs have been controversial since the first specimens were described 180 years ago[30,31]. Lydekker[32] included litopterns within "Ungulata", a wastebasket comprising fossil and extant ungulates generally, and in this he was followed by Osborn[33], Scott[1], and Schlosser[34]. Although Scott[1] presciently remarked on numerous resemblances between litopterns and perissodactyls, in the end he concluded that they were more likely the result of parallelism than actual close relationship. Ameghino[35], by contrast, claimed that it was "absolutely certain" that macraucheniids and other litopterns had a common origin with perissodactyls, distinct from the ancestry of other SANUs.

Simpson[36] viewed litopterns as directly derived from Condylarthra, a heterogeneous group of early Cenozoic mammals structurally and presumably phyletically ancestral to later, more advanced ungulates, and grouped them with Notoungulata, Astrapotheria, and Tubulidentata in a paraphyletic supraordinal entity, Protungulata. Subsequently, Simpson[2,37] suggested that all SANUs (including Pyrotheria and Xenungulata) evolved in South America from a North American condylarth ancestral stock, which arrived in South America during the Late Cretaceous or Early Paleocene. This scenario has been accepted by many later authors (*e.g.*, refs.[14, 18,19, 38, 39]).

McKenna[40,41] refined Simpson's proposal, contending that all SANUs (but not Tubulidentata) could be derived from a single North American condylarthran common ancestor. He proposed the name Meridiungulata for this putative monophyletic ensemble, but did not present an explicit cladistic analysis in support of it (see also ref. [42]).
Soria[3,43] also accepted a condylarthran origin for SANUs, but differed from McKenna in proposing a diphyletic origin for the latter. He argued that litopterns were explicitly related to certain North and South American condylarthrans and could thus be included within Protungulata (with content differing from Simpson's[36] concept). The remaining SANU orders were excluded from this grouping. A similar evolutionary scenario was proposed by Muizon and Cifelli[11], who coined the term Panameriungulata for a group including litopterns, South American didolodontids, and Kollpaniinae, an endemic subfamily of North American mioclaenids. The monophyly of Meridiungulata sensu McKenna[40,41] was also rejected by Tong and Lucas[44], Lucas[45], and Kondrashov and Lucas[46]. Horovitz[47] likewise found that Meridiungulata was paraphyletic, based on a cladistic study of postcranial elements. She concluded that litopterns and notoungulates were sister taxa, allied with meniscotheriid and phenacodontid condylarthrans, but separate from astrapotheres. In a similar vein, the preferred tree of placental cladistic relationships published by O´Leary *et al.*[48] positioned Litopterna (represented by *Protolipterna*) within stem Pan-Euungulata

while Notoungulata (represented by *Thomashuxleya*) grouped with Afrotheria, thereby denying once again the monophyly of Meridiungulata.

Muizon *et al.*[49] recently took up the question again, ruling in favor of meridiungulate monophyly but favoring close relationship with Artiodactyla. However, inasmuch as their data matrix did not include any perissodactyls, their analysis does not contradict the results of recent proteomic studies[50,51], which conclude that both litopterns (represented by *Macrauchenia*) and notoungulates (represented by *Toxodon*) are more closely related to Perissodactyla than to any other extant placental group (see also ref. [52]). In the absence of consensus in phenomic analyses, and lack of molecular information for other SANU orders, it remains unsettled whether all South American native ungulates are related monophyletically.

Supplementary Figure 7. Geographic distribution of Pleistocene *Macrauchenia patachonica* and closely related taxa *Xenorhinotherium bahiensis* and *Macraucheniopsis ensenadensis* (based on ref. 53; see also refs 54-61). The arrow indicates the locality of Cueva Baño Nuevo-1, the source of the sample of *Macrauchenia patachonica* (FACSO/BN-1/2A/5) utilized for this study (see Supplementary Figure 8).

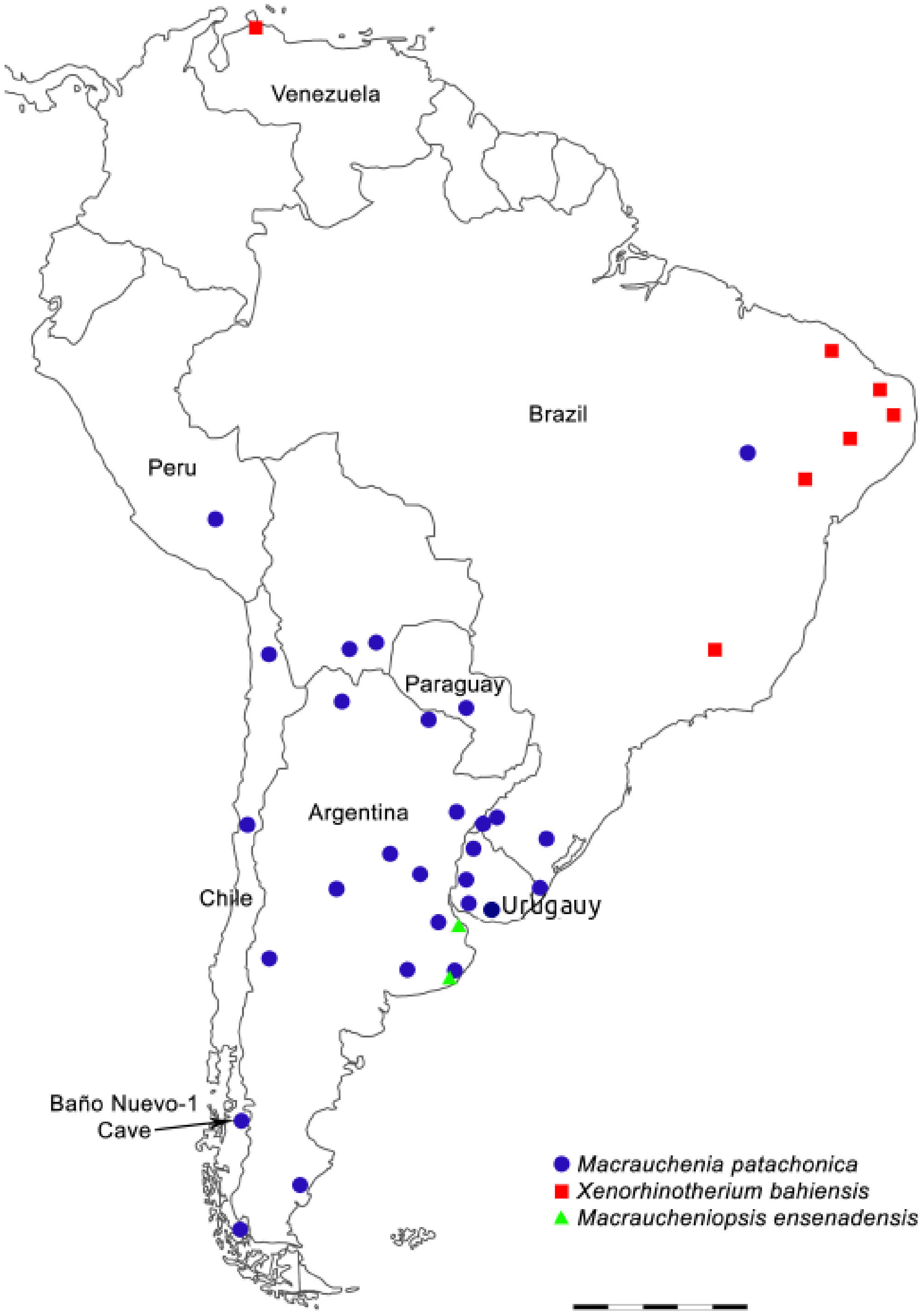

Supplementary Figure 8. Stratigraphic cross-section of Baño Nuevo-1 Cave, situated *ca.* 80 km NE of Coyhaique (45° 17′ S; 71° 32′ W), Región XI, Chile. The site is located within the Cerro Grande del Campo Seis, an Aptian (E. Cretaceous) volcanic complex. The cave has a depth of 20 m and an average width of 4 m. A middle phalanx of *Macrauchenia* (red star), designated in this study as MAC002 (and originally catalogued as fondecyt 1030560) was recovered from Layer 5 (clay and organic sands). This specimen yielded a date of 11,115 ± 30 ^{14}C yr BP (UCIAMS 166314), which is consistent with its stratigraphic position and association with faunal elements typical of late Pleistocene Patagonian faunas, including representatives of Ursidae, Equidae, Felidae, Camelidae, and Mylodontidae. *Macrauchenia* specimens were also recovered from overlaying Layer 4B[62].

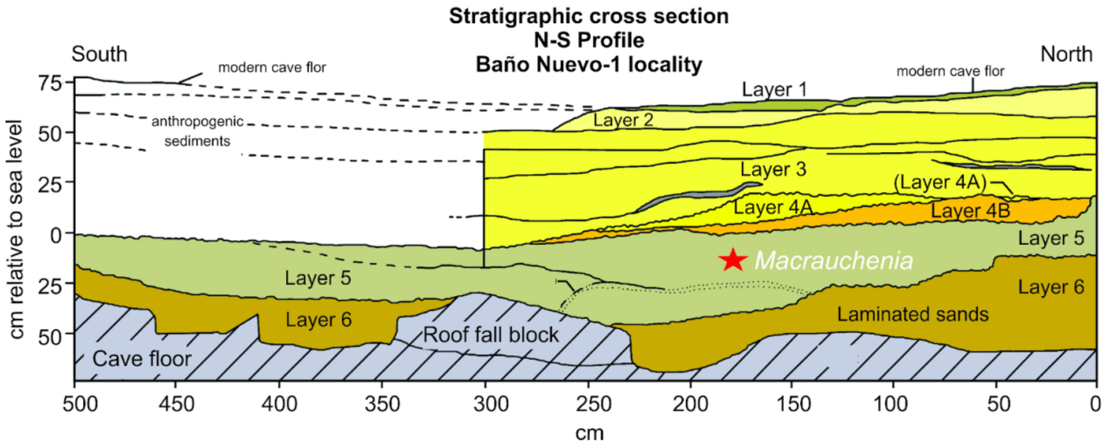

Supplementary References

1. Scott, W.B. Mammalia of the Santa Cruz beds. Part I. Litopterna. *Reports of the Princeton University Expedition to Patagonia* **7**, 1–156 (1910).
2. Simpson, G.G. *Splendid Isolation* (Yale Univ. Press, New Haven, 1980).
3. Soria, M.F. Los Proterotheriidae (Mammalia, Litopterna): sistemática, origen y filogenia. *Monogr. Mus. Argent. Cienc. Nat. "Bernardino Rivadavia"* **1**, 1–167 (2001).
4. Bond, M., Reguero, M.A., Vizcaíno, S.F. & Marenssi, S.A. in *Cretaceous-Tertiary high-latitude palaeoenvironments, James Ross Basin, Antarctica* (eds Francis J.E., Pirrie D., & Crame J.A.) 163–176. (Geological Society of London, 2006).
5. Reguero, M.A., Gelfo, J.N., López, G.M., Bond, M., Abello, A., Santillana, S.N. & Marenssi, S.A. Final Gondwana breakup: the Paleogene South American native ungulates and the demise of the South America–Antarctica land connection. *Global Planet. Change* **123**, 400–413 (2014).
6. Gelfo, J.N., Mörs T., Lorente, M., López, G.M. & Reguero, M. The oldest mammals from Antarctica, Early Eocene of the La Meseta Formation, Seymour Island. *Palaeontology* **58**, 101–110 (2015).
7. Cifelli, R.L. & Soria, M.F. Notes on Deseadan Macraucheniidae. *Ameghiniana* **20**, 141–153 (1983).
8. Bond, M. Quaternary native ungulates of Southern South America. A synthesis. *Quatern. South Amer. Antarc. Pen.* **12**, 177–205 (1999).
9. Scott, W.B. *A History of Land Mammals in the Western Hemisphere* (Macmillan, 1913).
10. Bonaparte, J.F. & Morales, J. Un primitivo Notonychopidae (Litopterna) del Paleoceno Inferior de Punta Peligro, Chubut Argentina. *Estud. Geol.* **53**, 263–274 (1997).
11. De Muizon, C. & Cifelli, R.L. The "condylarths" (archaic Ungulata, Mammalia) from the Early Palaeocene of Tiupampa (Bolivia): implications on the origin of the South American ungulates. *Geodiversitas* **22**, 47–150 (2000).
12. Soria, M.F. Notopterna: un nuevo orden de mamíferos ungulados eógenos de América del Sur. Parte I. Los Amilnedwardsidae. *Ameghiniana* **25**, 245–258 (1989).
13. Soria, M.F. Notopterna: un nuevo orden de mamíferos ungulados eógenos de América del Sur. Parte II. *Notonychops powelli* gen. et sp. nov. (Notonychopidae nov.) de la Formación Río Loro (Paleoceno medio, Provincia de Tucumán, Argentina). *Ameghiniana* **25**, 259–272 (1989).
14. Cifelli, R.L. in *Mammal phylogeny* (eds F.S. Szalay F.S., M.J. Novacek, N.J. & McKenna, M.C.) **2**, 195–216 (Springer, 1993).
15. Billet, G., Muizon, C., Schellhorn, R., Ruf, I., Ladevèze, S. & Bergqvist, L. Petrosal and inner ear anatomy and allometry amongst specimens referred to Litopterna (Placentalia). *Zool. Jour. Linn. Soc.* **173**, 956–987 (2015).
16. Gelfo, J.N., Ortiz-Jaureguizar, E. & Rougier, G.W. New remains and species of the 'condylarth' genus *Escribania* (Mammalia: Didolodontidae) from the Palaeocene of Patagonia, Argentina. *Trans. R. Soc. Edinburgh* **98**, 127–138 (2007).

17. Woodburne, M.O., Goin, F.J., Raigemborn, M.S., Heizler, M., Gelfo, J. N., Oliveira, E. V. Revised timing of the South American Early Paleogene land mammal ages. *J. South Am. Earth Sci.* **54**, 109–119 (2014).

18. Paula Couto, C. Fossil mammals from the beginning of the Cenozoic in Brazil. Condylarthra, Litopterna, Xenungulata and Astrapotheria. *Bull. Amer. Mus. Nat. Hist.* **99**, 359–394 (1952).

19. Cifelli, R.L. The origin and affinities of the South American Condylarthra and Early Tertiary Litopterna (Mammalia). *Amer. Mus. Novitates* **2772**, 1–49 (1983).

20. Bergqvist, L.P. Deciduous premolars of Paleocene litopterns of São José de Itaboraí Basin, Rio de Janeiro, Brazil. *Jour. Paleont.* **84**, 858–867 (2010).

21. Forasiepi, A.M., MacPhee, R.D.E., Hernández Del Pino, S., Schmidt, G.I., Amson, E. & Grohé, C. Exceptional skull of *Huayqueriana* (Mammalia, Litopterna, Macraucheniidae) from the Late Miocene of Argentina: anatomy, systematics, and paleobiological implications. *Bull. Amer. Mus. Nat. Hist.* **404**, 1–76 (2016).

22. Burmeister, G. Beschreibung der *Macrauchenia patachonica* Owen (*Opisthorhinus falkoneri* Brav.) nach A. Bravard´s Zeichnungen und den im Museo zu Buenos Aires vorhandenen Resten entworfen. *Abh. Naturforsch. Ges. Halle* **1**, 75–112 (1864).

23. Scott, W.B. *A History of Land Mammals in the Western Hemisphere* (MacMillan, 1937).

24. Rusconi, C. Evolución de la trompa en las macrauquenias. *Rev. Mus. His. Nat. Mendoza* **10**, 111–118 (1957).

25. Soria, M.F. Los Litopterna del Colhuehuapense (Oligoceno Tardío) de la Argentina. *Rev. Mus. Argent. Cienc. Nat. "Bernardino Rivadavia"* **3**, 1–54 (1981).

26. Ameghino, F. Apuntes preliminares sobre el género *Theosodon*. *Rev. Jard. Zool. Buenos Aires* **1**, 20–29 (1893).

27. Soria, M.F. in *Actas de IV Congreso Argentino de Paleontología y Bioestratigrafía*, 157–164 (1986).

28. Schmidt, G.I. & Ferrero, B.S. 2014. Taxonomic reinterpretation of *Theosodon hystatus* Cabrera and Kraglievich, 1931 (Litopterna, Macraucheniidae) and phylogenetic relationships of the family. *Jour. Vert. Paleon.* **34**, 1231–1238 (2014).

29. Tonni E.P. 1990. Mamíferos del Holoceno en la Provincia de Buenos Aires. *Paulacoutiana* **4**, 3–21.

30. Owen, R. A description of the cranium of *Toxodon platensis*, a gigantic extinct mammiferous species, referable by its dentition to the Rodentia, but with affinities to the Pachydermata and the herbivorous Cetacea. *Proc. Geol. Soc. London* **2**, 541–542 (1837).

31. Owen, R. Fossil Mammalia. In *The Zoology of the voyage of H.M.S. Beagle, Under the Command of Captain Fitzroy, during the Years 1832 to 1836* (ed. Darwin, C), **1**(1), 1–40 (Smith Elder, 1838).

32. Lydekker, R. Contributions to knowledge of the fossil vertebrates of Argentina, 3. A study of the extinct ungulates of Argentina. *An. Mus. La Plata* **2**, 1–91 (1893).

32. Osborn, H.F. *The Age of Mammals in Europe, Asia, and North America* (Macmillan, 1910).

34. Schlosser, M. in *Grundzüge der Paläontologie (Paläozoologie). II. Abteilung: Vertebrata* (ed. Zittel, K.A.) 402–689 (Oldenbourg, 1923).

35. Ameghino, F. Les formations sédimentaires du Crétacé Supérieur et du Tertiaire de Patagonie avec un parallèle entre leurs faunes mammalogiques et celles de l'ancien continent. *An. Mus. Nac. Buenos Aires* **8**, 1–568 (1906).

36. Simpson, G.G. The principles of classification and a classification of mammals. *Bull. Amer. Mus. Nat. Hist.* **85**, 1–350 (1945).

37. Simpson, G.G. The beginning of the age of mammals in South America. Part 1. Introduction. Systematics: Marsupialia, Edentata, Condylarthra, Litopterna and Notioprogonia. *Bull. Amer. Mus. Nat. Hist.* **91**, 1–232 (1948).

38. Reig, O.A. Teoría del origen y desarrollo de la fauna de mamíferos de América del Sur. *Publ. Mus. Mun. Cien. Nat. "Lorenzo Scaglia"* 1-162 (1981).

39. Cifelli, R.L. in *The Great American Biotic Interchange* (eds Stehli, F.G. & Webb, S.D.) 249–266 (Plenum, 1985).

40. McKenna M. C. in *Phylogeny of the Primates* (eds Luckett, W.P. & Szalay, F.S.) 21–46 (Plenum, 1975). Press, New York.

41. McKenna M.C. in *Evolutionary Biology of the New World Monkeys and Continental Drift* (eds Ciochon, R.L. & Chiarelli, A.B.) 43–77 (Plenum, 1981).

42. McKenna, M.C. & Bell, S.K. *Classification of Mammals above the Species Level* (Columbia Univ. Press, 1997).

43. Soria, M.F. Estudios sobre los *Astrapotheria* (Mammalia) del Paleoceno y Eoceno. Parte II: Filogenia, origen y relaciones. *Ameghiniana* **25**, 47-59 (1988).

44. Tong, Y. & Lucas, S.G. in *Proc. Third North Amer. Paleon. Conv.* **2**, 551–556 (1982).

45. Lucas, S..in *Mammal phylogeny* (eds F.S. Szalay F.S., M.J. Novacek, N.J. & McKenna, M.C.) **2**, 182–194 (Springer, 1993).

46. Kondrashov, P.E. & Lucas, S.G. *Palaeostylops iturus* from the Upper Paleocene of Mongolia and the status of Arctostylopida (Mammalia, Eutheria). *Bull. New Mexico Mus. Nat. Hist. Sci.* **26**, 195–203 (2004).

47. Horovitz, I. Eutherian mammal systematics and the origins of South American ungulates as based on postcranial osteology. *Bull. Carnegie Mus. Nat. Hist.* **36**, 63 –79 (2004).

48. O'Leary, M.A. *et al.* The placental mammal ancestor and the post-K-Pg radiation of placentals. *Science* **33**, 662–667 (2013).

49. De Muizon, C., Billet, G. Argot, C. Ladevèze, S. & Goussard, F. *Alcidedorbignya inopinata*, a basal pantodont (Placentalia, Mammalia) from the early Palaeocene of Bolivia: anatomy, phylogeny and palaeobiology. *Geodiversitas* **37**, 397–634 (2015).

50. Welker, F. *et al.* Ancient proteins resolve the evolutionary history of Darwin's South American ungulates. *Nature* **522**, 81–84 (2015).

51. Buckley, M. Ancient collagen reveals evolutionary history of the endemic South American "ungulates". *Proc. R. Soc.* **B282**, 20142671 (2015).

52. Beck, R.M.D. & Lee, M.S.Y. Ancient dates or accelerated rates? Morphological clocks and the antiquity of placental mammals. *Proc. R. Soc.* **281**, 20141278 (2014).

53. Scherer, C.S., Pitana V.G. & Ribeiro A.M. Proterotheriidae and Macraucheniidae (Litopterna, Mammalia) from the Pleistocene of Rio Grande Do Sul State, Brazil. *Rev. Brasil. Paleon.* **12**, 231–246 (2009).

54. Politis, G., Prado J.L., & Beukens, R. in *Ancient peoples and landscapes* (ed. Johnson, E.) 187–205 (Texas Tech Univ., 1995).

55. Panarello, H.O. & Fernández, J. Palaeoenvironmental changes in Leuto Caballo (Neuquén, Argentina) during Late Pleistocene - Holocene, evidenced by stable isotopes on marl and *Lymnaea*: first results. *An. Direc. Nac. Serv. Geol.* **34**, 418–421 (1999).

56. Ubilla, M. & Perea, D. Quaternary vertebrates of Uruguay: A biostratigraphic, biogeographic and climatic overview. *Quat. South Amer. Antarc. Pen.* **12**, 75–90 (1999).

57. Velásquez, H. & Mena, F. Distribuciones óseas de ungulados en la cueva Baño Nuevo 1 (XI Región, Chile): un primer acercamiento. *Magallania* **34**, 91–105 (2006).

58. López, P. & Labarca, R.O. *Macrauchenia* (Litopterna), *Hippidion* (Perissodactyla), Camelidae y Edentata en Calama (II Región): comentarios taxonómicos y tafonómicos. *Not. Mens. Mus. Nac. Hist. Nat.* **355**, 7–10 (2005).

59. Labarca, R.O. El Yacimiento paleontológico "Kamac Mayu": tafonomía y procesos de formación en el Cuaternario kárstico de la Cuenca de Calama (Región de Antofagasta-Chile). *Ameghiniana* **46**, 3–16 (2009).

60. Borrero, L.A. in *American Megafaunal Extinctions at the End of the Pleistocene* (ed. Haynes, G.)145–168 (Springer, 2009).

61. Tassara, D.A. and Cenizo, M.M. El patrimonio paleontológico en el sector costero al NE de Mar del Plata (Provincia de Buenos Aires, Argentina): Estado del conocimiento, vulnerabilidad y propuestas para su conservación. *Rev. Mus. Argent. Cienc. Nat. "Bernardino Rivadavia"* **16**, 165–183 (2014).

62. López, P., Mena, F. & Bostelmann, E. Presence of extinct bear in a pre-cultural level of Baño Nuevo-1 cave (Central Patagonia, Chile). *Estud. Geol.* **71**, e041 (2015).
